# Time-Series Clustering of lncRNA-mRNA Expression during the Adipogenic Transdifferentiation of Porcine Skeletal Muscle Satellite Cells

**DOI:** 10.3390/cimb44050138

**Published:** 2022-05-06

**Authors:** Xiaoyu Qiu, Guangliang Gao, Lei Du, Jing Wang, Qi Wang, Feiyun Yang, Xiaorong Zhou, Dingbiao Long, Jinxiu Huang, Zuohua Liu, Renli Qi

**Affiliations:** 1 Animal Nutrition Institute, Chongqing Academy of Animal Science, Chongqing 402460, China; qiuxiaoyu7004@sina.com (X.Q.); guanglianggaocq@hotmail.com (G.G.); wj57482199@163.com (J.W.); wangq0418@126.com (Q.W.); yfeiyun@yeah.net (F.Y.); longjuan880@163.com (D.L.); short00@163.com (J.H.); liuzuohua66@163.com (Z.L.); 2 Key Laboratory of Pig Industry Sciences, Ministry of Agriculture, Chongqing 402460, China; 3 State Key Laboratory of Animal Nutrition, College of Animal Science and Technology, China Agricultural University, Beijing 100193, China; dulei127899@163.com

**Keywords:** trans-differentiation, skeletal muscle satellite cells, lncRNA, transcriptome, temporal expression

## Abstract

Skeletal muscle satellite cells (SMSCs), which are multifunctional muscle-derived stem cells, can differentiate into adipocytes. Long-chain non-coding RNA (lncRNA) has diverse biological functions, including the regulation of gene expression, chromosome silencing, and nuclear transport. However, the regulatory roles and mechanism of lncRNA during adipogenic transdifferentiation in muscle cells have not been thoroughly investigated. Here, porcine SMSCs were isolated, cultured, and induced for adipogenic differentiation. The expressions of lncRNA and mRNA at different time points during transdifferentiation were analysed using RNA-seq analysis. In total, 1005 lncRNAs and 7671 mRNAs showed significant changes in expression at differential differentiation stages. Time-series expression analysis showed that the differentially expressed (DE) lncRNAs and mRNAs were clustered into 5 and 11 different profiles with different changes, respectively. GO, KEGG, and REACTOME enrichment analyses revealed that DE mRNAs with increased expressions during the trans-differentiation were mainly enriched in the pathways for lipid metabolism and fat cell differentiation. The genes with decreased expressions were mainly enriched in the regulation of cell cycle and genetic information processing. In addition, 1883 DE mRNAs were regulated by 193 DE lncRNAs, and these genes were related to the controlling in cell cycle mainly. Notably, three genes in the fatty acid binding protein (FABP) family significantly and continuously increased during trans-differentiation, and 15, 13, and 11 lncRNAs may target FABP3, FABP4, and FABP5 genes by cis- or trans-regulation, respectively. In conclusion, these studies identify a set of new potential regulator for adipogenesis and cell fate and help us in better understanding the molecular mechanisms of trans-differentiation.

## 1. Introduction

Trans-differentiation is a process through which the morphology and function of one type of cell change significantly to be transformed into another type of cell [1,2,3]. Skeletal muscle satellite cells (SMSCs) are located in the substrate of skeletal muscles and represent a committed stem cell population responsible for the postnatal growth and regeneration of skeletal muscle [4,5]. An increasing number of studies have shown that SMSCs and myoblasts can be transformed into adipocytes or adipose-like cells under certain conditions (drug stimulation and cytokine treatment) [6,7,8]. For example, a study has shown that the regeneration of mouse skin hair follicles was regularly accompanied by the transformation of a large number of muscle fibroblasts into ‘physiologically mature’ and ‘metabolically active’ white fat cells [9].

Several studies have investigated the molecular mechanism of trans-differentiation from muscle cells to adipocytes [10,11]. An in vitro experiment has shown that dexamethasone treatment induced trans-differentiation of rat tendon stem cells into adipocytes, which was accompanied by a significant increase in *DKK1* gene expression. Knocking out *DKK1* significantly inhibits adipogenesis [12]. Clearly, studying and understanding how muscle cells enter the adipogenic trans-differentiation program and what metabolic and genetic changes are induced are important for the correct interpretation of muscle tissue development and function. Therefore, the study about the SMSCs trans-differentiation is helpful for the diagnosis study and the treatment of muscle diseases and metabolic imbalance. Meanwhile, it would also provide a theoretical basis to control intramuscular fat content and improve pork quality.

Non-coding RNAs (ncRNAs), including microRNAs (miRNAs), long non-coding RNA (lncRNAs), and circRNAs, show complex and strong regulatory functions in the different physiological processes of cell proliferation, differentiation, and apoptosis [13,14,15]. It has been noted that some ncRNAs have been proved to play important roles in the control of muscle growth and development. For example, miRNA-181a affects muscle development in mice by targeting lncRNA gene Lnc-Malat1, which activates MYOD, the key transcription factor for genetic programming in myogenic differentiation [16]. Lnc-RAM and lncRNA genes directly bind to MYOD to regulate the expression of myogenic genes and ultimately affects the differentiation of myoblasts [17].

However, detailed studies to explore the role of ncRNAs in the adipogenic differentiation of muscle cells are sufficient. Our previous study indicated that miR-199a strongly regulates adipogenesis in C2C12 myoblasts by targeting FATP1 [13]. Moreover, we analysed and compared the differences in the expression profiles of lncRNAs between myogenic and adipogenic differentiation in C2C12 myoblasts using high-throughput sequencing [15]. LncRNA-GM43652 is highly expressed during adipogenesis, and the knockdown of this gene effectively inhibits trans-differentiation. These findings not only reveal the complexity of the functions of ncRNAs but also suggest that we need to more carefully and thoroughly assess the changes and functions of ncRNAs in the adipogenic trans-differentiation of muscle cells.

In the present study, we utilize RNA-seq and gene temporal expression analysis to reflect the dynamic changes of lncRNAs and mRNAs during the process of adipogenesis in SMSCs to find the key driver molecules for the control of adipogenesis and cellular fate.

## 2. Materials and Methods

### 2.1. Isolation, Culture, and Identification of Porcine SMSCs

The isolation, culture, and identification of SMSC were performed according to the Musarò’s method [18]. RongChang piglet, a characteristic Chinese endemic pig breed with high intramuscular fat content, was used in this study. Three heathy piglets less than 3 days of age were selected for the cell isolation. After exsanguination, 1 cm^3^ muscle blocks were cut from the middle of the longissimus and semitendinosus muscles and washed with precooled PBS (including 100 U/mL penicillin/streptomycin; GIBCO, Grand Island, NY, USA) three times. The connective tissue was cut in PBS and then quickly cut into 1 mm^3^ pieces and collected in a sterile centrifuge tube. Type I collagen 0.2% (Solarbio, Shanghai, China) was used to digest the tissue on a shaking table at 37 °C for 90 min. The samples were digested with 0.25% trypsin (Solarbio, Shanghai, China) for 30 min. The digestion was terminated with an equal volume of complete medium (10% fetal calf serum, DMEM-high sugar and 100U/mL penicillin/streptomycin; GIBCO, Grand Island, NY, USA) and passed through 100 um and 40 um cell sieves. The cells were re-suspended and inoculated into a 60 mm cell culture dish. After incubation in a CO_2_ incubator for 2 h, the supernatant was aspirated and the cell pellet was transferred to a new Petri dish and recorded as P01. P01 cells were cultured for 24 h and then suspended into the supernatant, which was recorded as P02. P02 was purified from SMSCs. The medium was changed for the first time after 48 h of culture, and then the complete culture medium was changed every two days.

SMSCs were inoculated into 24-well cell culture plates, and immunofluorescence staining of PAX 7 protein was performed when cell density reached 50%. After rinsing the cells with PBS three times, they were fixed with 4% paraformaldehyde for 30 min and rinsed with distilled water three times for 5 min each. Triton X-100 (0.1%) was allowed to permeate the cells for 30 min and then they were washed three times with distilled water for 5 min each time. 2% Goat serum (Solarbio, Shanghai, China) was used for blocking for 1 h. The primary antibody (anti Pax7, 1:300; Proteintech, Wuhan, China) was added to cover the bottom surface, and it was incubated at 4 °C overnight. After removing the primary antibody, the cells were washed with distilled water and incubated with the fluorescent secondary antibody (sheep anti-mouse, 1:100; Proteintech, Wuhan, China) at room temperature in the dark for 1 h. After absorbing the secondary antibody, 250 μL DAPI solution (Solarbio, Shanghai, China) was added and observed under a fluorescence microscope.

### 2.2. Adipogenesis Induction and Sample Collection

The adipogenic differentiation of SMSCs was based on our previous study, and improved adipogenic differentiation was induced at a cell density of 90%. First, the cells were cultured in DMEM-F12 medium (containing 10% foetal bovine serum, 850 nmol insulin, 1 µmol dexamethasone, 0.5 mmol 3-isobutyl-1-methylxanthine, and 1 µmol rosiglitazone; GIBCO, Grand Island, NY, USA) for 2 days (step 1). The cells were then cultured in medium containing insulin (850 nmol) and rosiglitazone (1 µmol/L) for another 2 days (Step 2). Steps 1 and 2 were recycled once, and the ordinary maintenance medium was replaced for culture cells for 2–4 days, and the solution was changed once every 2 days. The morphological changes and intracellular fat deposition of adipogenic differentiation cells were observed by Oil Red O staining solution (Solarbio, Shanghai, China). The cells were harvested at the undifferentiated (P), early (2 days), middle (4 days), and late (10 days) stages of lipogenic differentiation for RNA extraction. Three duplicate cell samples were collected from each time point.

### 2.3. RNA Sequencing

Briefly, total RNA was extracted from different groups of cell samples using the Trizol reagent (Invitrogen Life Technologies, Carlsbad, CA, USA). After determining for total RNA integrity and DNA contamination, we prepared the final cDNA library in accordance with a strand-specific library preparation by dUTP method. Then, the Illumina HISeq 2500 (Majorbio Biotechnology, Shanghai, China) platform was used to sequence the paired-end library. After removing low-quality reads, the clean reads were subjected to quality assessment. These included the classification of total and distinct reads and the assessment of their percentage in the library, analysis of saturation of the library, and correlation analysis of biological replicates. TopHat2 software was used to map the clean data to the porcine reference genome (Sus scrofa v.11.1, ftp://ftp.ncbi.nlm.nih.gov/genomes/Sus_scrofa, accessed on 13 April 2021). The number of perfect clean reads corresponding to each gene was calculated and normalized to the number of reads of Transcripts Per Million (TPM).

### 2.4. STEM Analysis and Enrichment Analysis

The short time-series expression miner (STEM) clustering algorithm [19] was used to identify temporal gene expression profiles during the lipogenic trans-differentiation of SMSC. First, representative temporal expression profiles were defined as model profiles, which were independent from the sequencing data. Then, the values of the gene expression were transformed to log ratios relative to the expression at the stage of P. Each gene was assigned to the filtering criteria of the model profiles, and the correlation coefficient was determined. The true ordering of time points was used to test the standard hypothesis according the model profile and the expected number of assigned genes to determine the *p*-value (adjusted *p*-value, 0.05 by Bonferroni correction).

Gene Ontology (GO), Kyoto Encyclopedia of Genes and Genomes (KEGG), and REACTOME were used to understand the regulatory functions of mRNAs in the 11 significantly enriched profiles, as previously described [20]. The enriched functions or pathways with *p adjust* < 0.05 or *q value* < 0.05 were identified as statistically significant.

### 2.5. Protein-Protein Interaction (PPI) Network Construction

The Search Tool for the Retrieval of Interacting Genes (STRING) (http://string-db.org, accessed on 28 April 2022) is a database for searching interactions that can show the direct physical interaction and the indirect functional correlation between proteins. We employed STRING database 25 to identify pairwise relationships among the DE mRNAs with up-regulated expressions in profile 1, 2, and 11 and the DE mRNAs with down-regulated expressions in profile 4 and 7, respectively, and then a protein–protein interaction (PPI) network was built.

### 2.6. Target Gene s of DE lncRNAs-mRNA

Two regulation models (cis-regulation and trans-regulation) were used to analyze the target genes of DE lncRNAs. For cis-regulation analysis, we predicted the target coding genes within 10 kb upstream and downstream of lncRNA; for the trans-regulation analysis, the target genes were predicted based on the free energy that is needed to form secondary structures between lncRNAs and mRNA sequences. RNAplex software (http://www.tbi.univie.ac.at/software, accessed on 20 May 2021) was used to predict potential target genes of lncRNAs.

### 2.7. Quantitative Real-Time PCR

We extracted total RNA using the Trizol reagent and carried out quantitative real-time PCR (qRT-PCR) in triplicate using the SYBR Premix Ex Taq II (TaKaRa Biotechnology, Shanghai, China). The mean expression values were calculated relative to the mean expression level of the housekeeping gene GAPDH. All of the reactions were prepared while using three replicates and the expression levels of genes were expressed as fold change using the 2^−∆∆CT^ method. Primer sequences are showed in Supplementary Table S1.

### 2.8. Statistical Analysis

The data were processed while using R, Microsoft Excel, and GraphPad Prism 8.5. Differences of gene expression among groups were analyzed using the Kruskal–Wallis test, and a false discovery rate (FDR) < 0.05 was considered to be statistically significant. In the case of qRT-PCR validation of the transcriptome, statistical analysis was carried out using GraphPad Prism 8.5 Software using one-way ANOVA. *p* < 0.05 was considered significant.

## 3. Results

### 3.1. Adipogenic Transdifferentiation of SMSCs

More than 90% of the cells obtained from the muscle of piglets were Pax7 positive, which is consistent with the identity of skeletal muscle satellite cells (Supplementary Figure S1A). These cells have a strong myogenic differentiation capability and can develop into multinucleated muscle cells under appropriate conditions (Supplementary Figure S1B).

**Figure 1:**
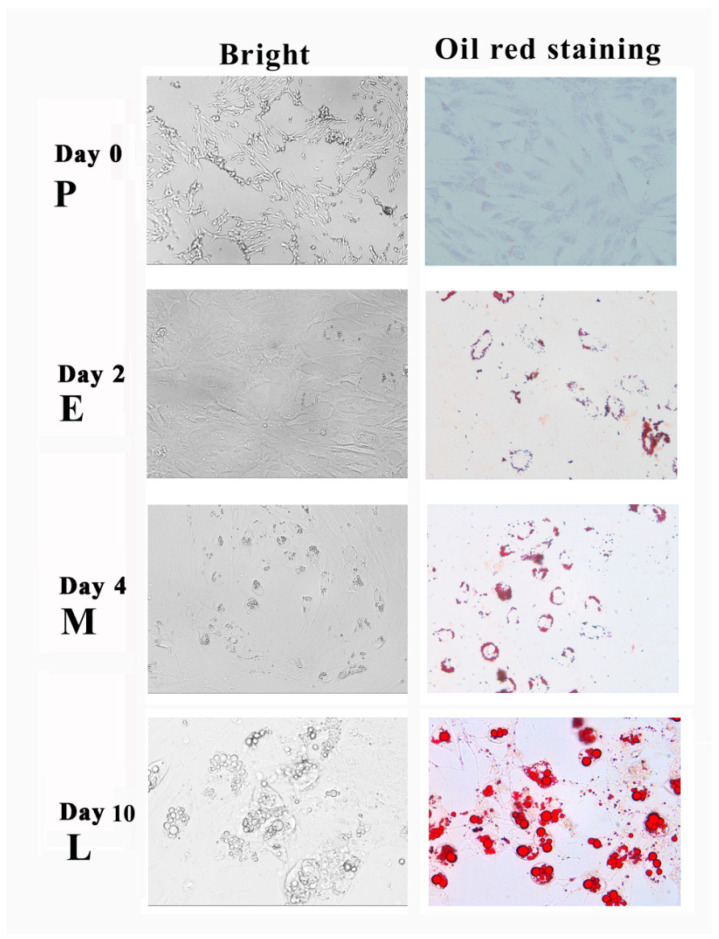
Adipogenic trans-differentiated porcine skeletal muscle satellite cells at pre-, early-, middle- and late-stages of differentiation. The cells are stained with Oil red O during the adipogenesis process.

Importantly, SMSCs also showed strong trans-differentiation abilities with the stimulation of adipogenic induction. A large number of lipid droplets began to be generated in the cells on the 2nd day of lipogenesis induction and formed large lipid droplets at the 10th day, which is shown by oil-red staining (Figure 1).

### 3.2. Summary of Sequencing and Gene Expression Analysis

The cells at the pre-differentiation (day 0, group P), early differentiation (day 2, group E), middle differentiation (day 4, group M), and later differentiation (day 8, group L) stages were collected and sequenced using the Illumina HiSeq 2000 sequencing platform. The average value for each sample was approximately 10.02 G. The Q20 of the sequencing data was more than 98%, and that of Q30 was more than 95%. After quality control of the original data and the removal of the reads compared to ribosomal RNA, an average of 95% of the clean reads was compared to the reference genome, of which the unique clean reads compared to the genome were more than 88% (Supplementary Table S2). These data showed that sequencing quality was good and satisfied the requirements for subsequent analysis.

A total of 6891 lncRNAs (6731 known genes and 160 new genes) were identified by sequencing, corresponding to 16,421 transcripts, with an average of 2.38 transcripts per gene. A total of 21,478 mRNA genes (21,365 known genes and 113 new genes) were identified, corresponding to 57,592 transcripts, with an average of 2.68 transcripts per gene.

As shown in Supplementary Figure S2A, the expression levels of lncRNAs and mRNAs were not significantly different among the four groups of cells. However, the mRNA expression level was higher than that of lncRNA. Principal component analysis (PCA) and sample correlation analysis showed that the gene expression patterns of pre-differentiated cells (group P) were significantly different from those of adipogenic differentiated cells (groups E, M, and L). The cells in groups M and L showed similar gene expression levels (Supplementary Figure S2B,C).

The top 20 most highly expressed lncRNA genes in each group of cells are shown in Supplementary Table S3. ENSSSCG00000051102, ENSSSCG00000048719, MSTRG.23453, ENSSSCG00000041875, and ENSSSCG00000041401 were highly expressed in all four cell types. With the exception of ENSSSCG00000041875, the expression of the other four genes gradually increased during adipogenic differentiation. Among the top 20, the expression of ENSSSCG00000045913, ENSSSCG00000050010, and MSTRG.20538 decreased gradually during adipogenic differentiation. The top 20 most highly expressed mRNA genes are shown in Supplementary Table S4.

**Figure 2:**
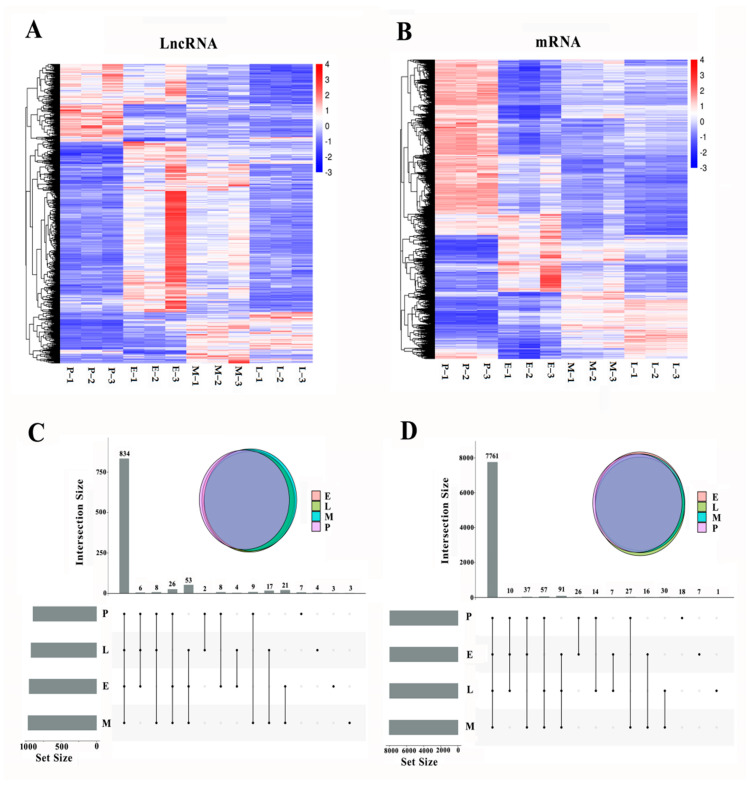
Differential expressed (DE) lncRNA and mRNA. (**A**). The clustered Heatmap of the global expression changes of DE lncRNAs. Red indicates high expression levels, and blue represents low expression levels. (**B**). The clustered Heatmap of the global expression changes of DE mRNAs. (**C**). The Upset Venn of total amount of the unique lncRNAs in different stages of the cells. (**D**). The Upset Venn of total amount of the unique mRNAs in different stages of the cells.

Differences in the expressions of lncRNAs and mRNAs between the four groups of cells were analyzed using the Kruskal–Wallis test. A total of 1005 lncRNAs showed significant changes on the expression at four stages (FDR < 0.05). Similarly, 7671 mRNA were significantly differentially expressed with time (FDR < 0.05). The clustered heatmaps in Figure 2A,B show the global expression profiles of differentially expressed (DE) lncRNAs and mRNAs. The Upset Venn figure shows the total number of unique lncRNAs (Figure 2C) and mRNA (Figure 2D) in different groups of cells.

### 3.3. Temporal Gene Expression Patterns of DE lncRNAs and DE mRNAs

**Figure 3:**
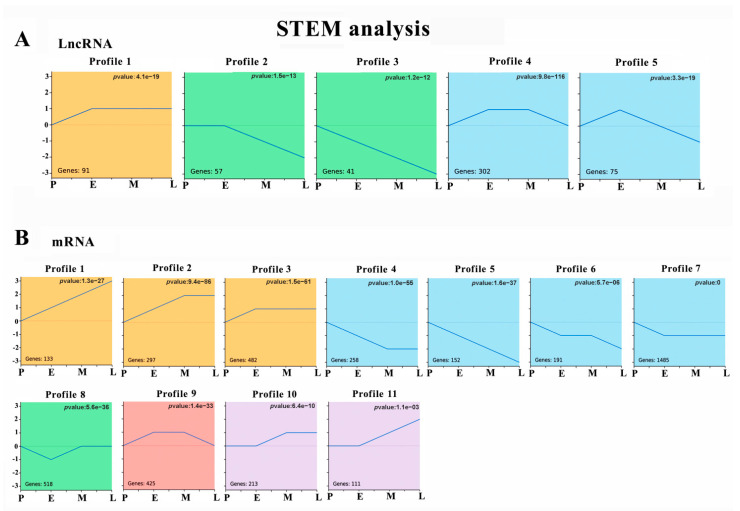
STEM identified the temporal expression profiles of DE lncRNA and DE mRNAs with *p* < 0.05: (**A**) 5 clustered profiles of temporal expressed DE lncRNA; (**B**) 11 clustered profiles of temporal expressed DE mRNA. The blue lines in the profile boxes depict the gene expression patterns at the four time points. The number on the bottom left represents the number of genes and the number on the top right corner represents the adjusted *p*-value.

We normalised the sequencing data to the samples in group P and then identified the temporal gene expression profiles using STEM. As shown in Figure 3A, 566 of the 1005 DE lncRNAs clustered into five profiles with *p* *adjust* < 0.05. Modules with the same colour in Figure 3 indicate that the gene expression trends were relatively similar. Among them, 91 DE lncRNAs in profile 1 were stably and highly expressed after adipogenesis induction. Meanwhile, 57 and 41 DE lncRNAs in profile 2 and profile 3 were downregulated during lipogenesis. Notably, Profile 3 showed that lncRNA expression was continuously downregulated at all four time points. Profile 4 revealed 302 DE lncRNAs highly expressed at stages E and M, and profile 5 showed that 75 DE lncRNAs were mainly upregulated at stages E mainly.

As shown in Figure 3B, STEM analysis classified 4265 DE mRNAs into 11 profiles (*p* < 0.05). Profiles 1 (133 genes), 2 (297 genes), 3 (482 genes), 10 (213 genes), and 11 (111 genes) showed that DE mRNAs were upregulated during lipogenic differentiation. Meanwhile, profiles 4 (258 genes), 5 (152 genes), 6 (191 genes), and 7 (1485 genes) showed that DE mRNAs were downregulated during the differentiation. Profile 8 revealed 518 DE genes with low expression at stage E, and profile 9 showed 425 DE mRNAs with upregulated expression in the E and M groups of cells. Notably, profile 1 and profile 5 showed that 133 and 155 DE mRNAs were continuously upregulated or downregulated at all four time points, respectively. These temporal expression analysis results provided effective information for further identifying the core factors driving adipogenesis in muscle cells.

### 3.4. GO and KEGG Analysis of the Temporal Expressed DE mRNAs

**Figure 4:**
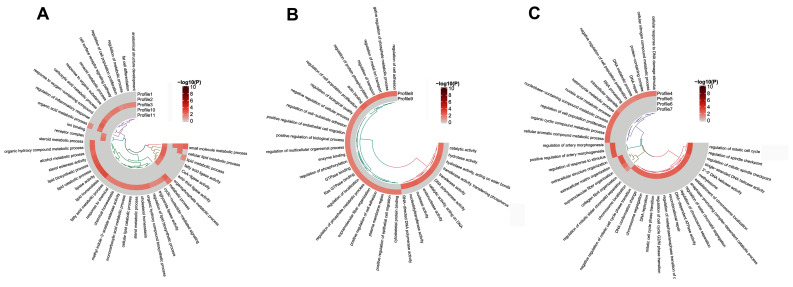
GO analysis of temporal expressed DE mRNAs. (**A**) Significant enriched GO terms for up-regulated genes during the trans-differentiation. (**B**). Significant enriched GO terms for genes showed changes in the differentiated midsection. (**C**). Significant enriched GO terms for down-regulated genes during the trans-differentiation.

GO functional enrichment analysis was performed for the DE mRNAs in the 11 temporal gene expression profiles. The results were shown in Figure 4. The results indicated that the upregulated mRNAs (clustered in profiles 1, 2, 3, 10, and 11) were enriched in GO terms involved in lipid metabolism mainly. In particular, the genes contained in profiles 1, 2, and 11 were significantly enriched in multiple terms related to lipid metabolism, such as cellular lipid metabolism process, fatty acids ligase activity, lipid catabolic process, lipid biosynthetic process, and fat cell differentiation. All genes in profiles 1, 2, and 11 are listed in Supplementary Table S5.

In Figure 4B, the mRNAs in profile 8 and 9 showed functions in the regulation of cell adhesion, regulation of transport, regulation of protein phosphorylation, and nuclease activity, etc. It was observed that mRNAs with down-regulated expression during the trans-differentiation (clustered in profiles 4, 5, 6, and 7) showed functions in the regulation of nucleic acid metabolism, DNA metabolism and repair, cell proliferation regulation, and cell cycle regulation mainly (Figure 4C). In particular, the genes in profiles 4 and 7 showed the regulation for DNA metabolism, RNA metabolism, and cell cycle, and so these genes may be responsible for the changes in cell fate primarily. All genes in profiles 4 and 7 are listed in Supplementary Table S6.

**Figure 5:**
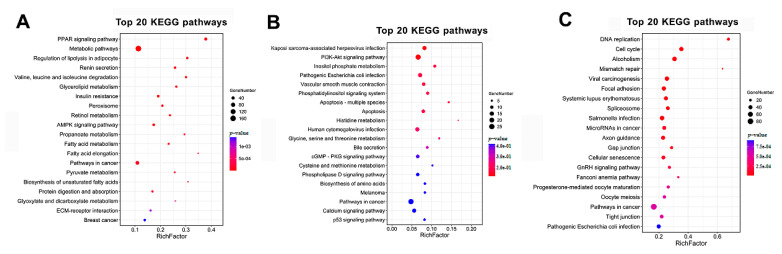
KEGG enrichment analysis of temporal expressed DE mRNAs. (**A**) Significant enriched KEGG pathways for the up-regulated genes during the trans-differentiation. (**B**) Significant enriched KEGG pathways for genes showed changes in the differentiated midsection. (**C**) Significant enriched KEGG pathways for the down-regulated genes during trans-differentiation.

Next, the KEGG analysis showed metabolic pathway enrichment for the altered genes that revealed the changes in cell fate and cellular metabolism mediated by DE mRNAs. The top three pathways enriched by the upregulated mRNAs in profiles 1, 2, 3, 10, and 11 were PPAR signalling pathway, fatty acid elongation, regulation of lipolysis in adipocyte (Figure 5A). The genes in profiles 8 and 9 were enriched the pathways including histidine metabolism, apoptosis, glycine, serine, and threonine metabolism (Figure 5B). The top three pathways enriched by the down-regulated mRNAs in profiles 4, 5, 6, and 7 were DNA replication, mismatch repair, and cell cycle (Figure 5A).

### 3.5. REACTOME Pathways Analysis of the Temporal Expressed DE mRNAs

**Figure 6:**
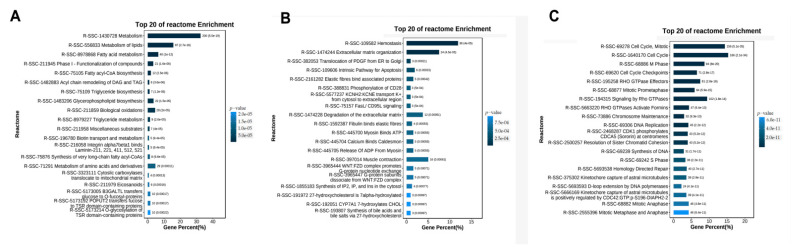
REACTOME pathway enrichment analysis of temporal expressed DE mRNAs. (**A**) Top 20 enriched REACTOME pathways for the up-regulated genes during trans-differentiation. (**B**) Top 20 enriched REACTOME pathways for the genes showed changes in the differentiated midsection. (**C**) Top 20 enriched REACTOME pathways for the down-regulated genes during trans-differentiation.

As a free, open-source, and peer-reviewed knowledge-base of biomolecular pathway analysis methods, REACTOME provide bioinformatics tools for visualization, interpretation, and analysis of pathway knowledge to support basic research, genome analysis, modelling, and systems biology [20]. REACTOME analysis was also enlisted to analyze and visualize the pathways involved in the temporal expressed DE mRNAs. The top five REACTOME pathways enriched by the upregulated mRNAs in profiles 1, 2, 3, 10, and 11 were metabolism, metabolism of lipids, fatty acids metabolism, phase I-functionalization of compounds, and fatty acyl-CoA biosynthesis (Figure 6A). The genes in profiles 8 and 9 were enriched top five REACTOME pathways including hemostasis, extracellular matrix organization, translocation of PDGF from ER to Golgi, intrinsic pathway for apoptosis (Figure 6B). The top five REACTOME pathways enriched by the upregulated mRNAs in profiles 4, 5, 6, and 7 were cell cycle mitotic, cell cycle, M Phase, cell cycle checkpoint, and RHO GTPase effectors (Figure 6C).

### 3.6. Protein–Protein Interaction among Temporal Expressed DE mRNAs Related to Lipid Metabolism and Cell Cycle

**Figure 7:**
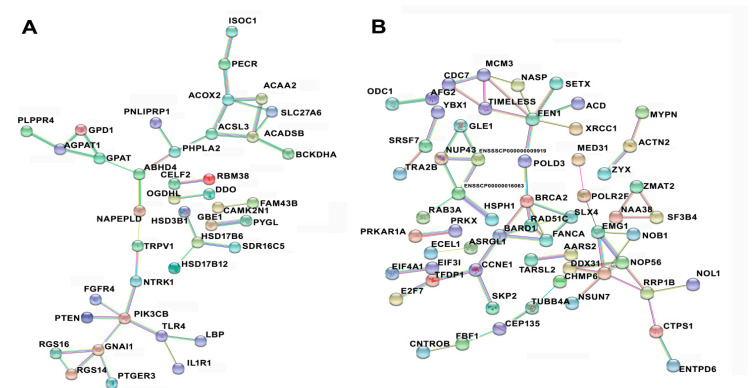
Protein–protein interaction network of differentially expressed mRNAs. (**A**) Probable functional network of mRNAs in profiles 1, 2, and 11. These genes were related to the regulation for lipid metabolism. (**B**) Probable functional network of the mRNAs in profiles 4 and 7. These genes were related to the regulation for cell cycle and genetic information processing.

By conducting GO, KEGG, and REACTOME enrichment analyses, lipid metabolism and cell cycle-related DE mRNAs were selected for protein–protein interaction (PPI) network analysis. The network was constructed using the STRING database. Figure 7A shows the PPI network comprising the genes in profiles 1, 2, and 11. These genes with increases in expression during trans-differentiation function for regulating lipid metabolism mainly. It had been observed that ABHD4, ACSL3, PIK3CB, ACOX2, and were major node molecule in the interaction net. Figure 7B shows the PPI network comprising genes in profiles 4 and 7. It had been observed that FEN1, BARD1, CCNE1, RAD51C, and TIMELESS were major node molecule in the interaction net. These genes with decreases in expression during trans-differentiation function for regulating cell cycle and DNA metabolism.

### 3.7. The Targeted mRNAs of DE lncRNAs

**Figure 8:**
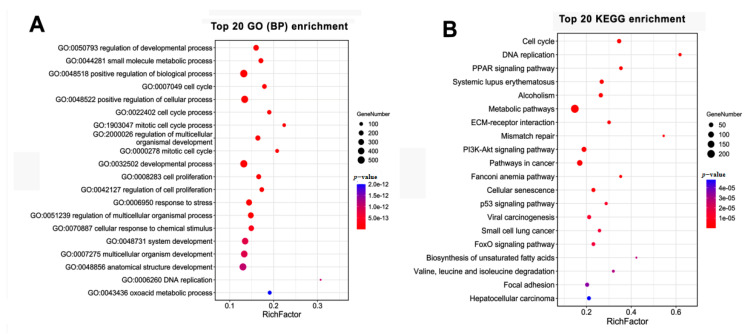
GO and KEGG enrichment analysis of the DE mRNAs targeted by DE LncRNAs. (**A**) Top 20 GO enrichment terms (biological process). (**B**) Top 20 KEGG enrichment pathways.

Out of the 7671 DE mRNAs, 1884 were regulated by 193 in the 1005 DE lncRNAs that were screened by target predication analysis. There were a total of 19,860 targeting relationships between DE lncRNAs and DE RNAs including 21 cis-regulations and 19,839 trans-regulations. Furthermore, the DE 1884 mRNAs were analysed by GO and KEGG to ascertain their functions and related pathways (Figure 8). The top five enriched GO terms were DNA replication, mitotic cell cycle process, mitotic cell cycle, cell cycle process, and cell cycle. The top five enriched KEGG pathways were DNA replication, mismatch repair, biosynthesis of unsaturated fatty acids, PPAR signalling pathway, and cell cycle. From the results, it has been observed that the most significant changes on cell cycle and genetic information processing are mediated by lncRNA-mRNA interactions.

### 3.8. Expressions of FABP Family Genes

**Figure 9:**
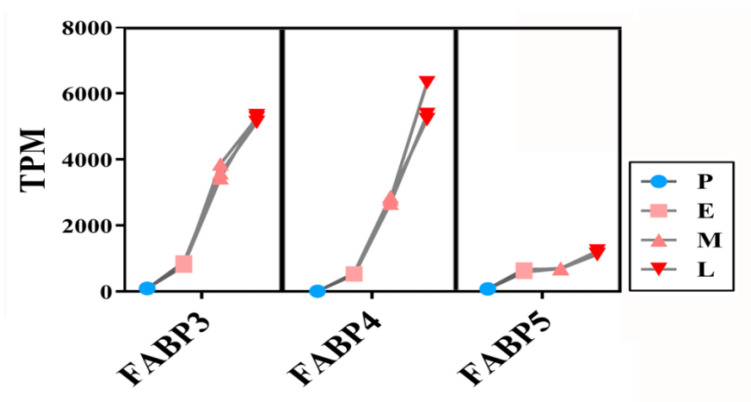
Expression changes of FABP genes during the lipogenesis of SMSC. Sequencing data, *n* = 3.

Among the mRNAs upregulated during the adipogenic differentiation of SMSCs, we particularly noticed fatty acid binding protein (FABP) family genes. Previous studies have reported that FABP genes (especially FABP4) participate in the regulation of lipid synthesis and adipocyte differentiation [21], and their expression level is positively correlated with intramuscular fat deposition [22]. Our sequencing data showed that FABP3, FABP4, and FABP5 were continuously highly expressed in the middle stage (M group) and late stage (L group) of adipogenic trans-differentiation (Figure 9). FABP4 expression was the third highest among all of the mRNAs in the late stage (L group). These results suggest that high FABP expression promotes the production and deposition of lipid droplets during adipogenic differentiation of muscle cells, especially at a later stage. STEM analysis also showed that FABP3 and FABP4 clustered in profile 2 and FABP5 clustered in profile 3.

**Figure 10:**
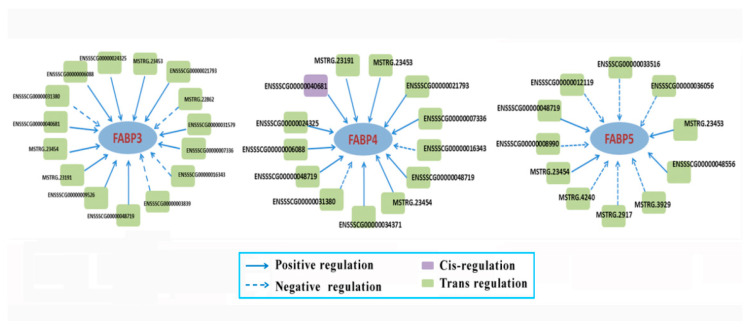
The lncRNAs targeted FABP genes by cis- or tans-regulation.

We also screened DE lncRNAs for targeting the three FABP genes especially. 11, 13, and 15, lncRNAs were obtained by predicting the potentially regulated FABP3, FABP4, and FABP5, respectively (Figure 10). Among them, only the relationship between ENSSSCG00000040681 and FABP4 was cis-regulation, while the others were trans-regulation.

### 3.9. Verification of the Reliability of Sequencing Results

**Figure 11:**
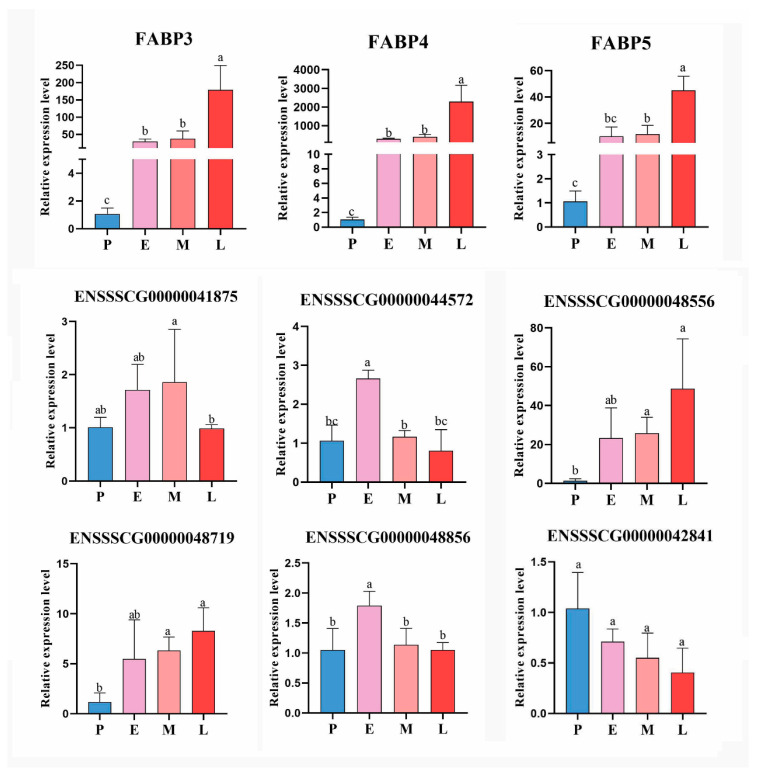
qPCR validation of the RNA-Seq expression results of three FABP genes and six DE lncRNAs. The data are presented as the means ± SEM. *n* = 3. Groups with the same letters have no difference with *p* > 0.05, and groups with different letters have difference with *p* < 0.05.

Next, to validate RNA-seq results, three FABP genes and six other DE lncRNAs were selected for qRT-PCR. As shown in Figure 11, qPCR data for the expression of selected genes coincided with the sequencing data, thereby indicating that our transcript identification and abundance estimation are highly reliable.

## 4. Discussion

LncRNA is a type of non-coding RNA molecule with a transcript length of more than 200 nt. It widely exists in various tissues of organisms [23,24,25]. With the discovery of lncRNA function, the important role of lncRNAs in different biological process has been gradually realised. The differentiation of muscle cells was a complex biological process regulated by many signal pathways, transcription factors, and functional genes [3,26,27,28,29]. The functions and mechanisms of a small number of lncRNAs in the control of physiological homeostasis in muscle tissue have been clearly established in previous studies, including MEG3, LncMD, Lnc113b, LNC-SEMT, LNC-Six1, and LncIRS1 [30,31,32,33,34,35,36,37]. Clearly, the roles of lncRNAs in the growth, development, and disease formation of muscle tissue cannot be ignored and need to be explored.

As muscle-derived pluripotent stem cells, SMSCs and muscle precursor cells can produce and store a large number of lipids and differentiate eventually into fat cells under special circumstances [38]. This adipogenic ability was strengthened by rosiglitazone, an agonist of PPARgama, the main promoter of cellular adipogenesis [39]. Other stimuli, such as high glucose and fatty acids, can also cause muscle cells to produce lipids to varying degrees [40]. The SMSCs in adipogenic differentiation change cell morphology and metabolism, which requires the expression changes of multiple factors to be addressed by different signalling pathways. Our previous study also reported beneficial results. We identified 114 core lncRNAs that showed significant differences in the expression between myogenesis and adipogenesis in C2C12 muscle precursor cells. The expression of lncRNA-GM43652 increased significantly during adipogenic differentiation, and we confirmed that lncRNA-GM43652 had a significant effect on cellular lipid deposition following the gene knockout [15]. These findings underscore the importance of lncRNAs in the adipogenic trans-differentiation of muscle cells; however, the results are limited because the investigation of lncRNA expression did not cover the entire process of adipogenesis.

During SMSC adipogenesis, the timing of gene expression is important, and gene expression at a single time point is insufficient to fully understand the dynamic characteristics of gene expression in the adipogenic differentiation of SMSCs. Therefore, in this study, we have analysed and compared the expression profiles of lncRNAs and mRNAs at different time points during adipogenesis using STEM temporal expression analysis to identify genes that are important for the regulation of trans-differentiation. We identified 1005 DE lncRNAs and 7671 DE mRNAs with altered expression during lipogenesis, in which DE 566 lncRNAs and 4265 DE mRNAs were clustered into 5 and 11 profiles, respectively, with *p* < 0.05, by STEM analysis. In different clustered profiles, lncRNAs and mRNAs showed significant increases or decreases in expression at different time stages. These altered genes play pivotal regulatory roles at the differential stages of trans-differentiation.

The functions and related pathways of the altered genes were analyzed and contoured by bioinformatics tools, and we obtained some important information about the changes in genetic programming for shifting cell fate and cellular metabolism. The up-regulated mRNAs, especially hundreds of genes in profile 1, 2, and 11, were remarkably related to the control of lipid metabolism and fat cell differentiation, such as ABHD4, ACSL3, PIK3CB, and ACOX2. The down-regulated mRNAs in profile 4 and 7 were mainly enriched in the regulation for cell cycle, nucleic acid metabolism, and cell proliferation, such as FEN1, BARD1, CCNE1, RAD51C, and TIMELESS. Clearly, these genes may be responsible for changes in muscle cell fate primarily. By subsequent target gene prediction analysis, more than 20,000 cis- and trans-targeting relationships were found between DE lncRNAs and DE mRNAs. This also reflects the complexity of molecular networks involved in the control of trans-differentiation.

In addition, we also analyzed the overall interaction between DE lncRNAs and DE mRNAs. Total 19,860 targeting relationships between DE lncRNAs and DE RNAs were found by target predication and the most significant changes on cell cycle and genetic information processing that are mediated by the lncRNA-mRNA interaction. This certainly reveals the important and complex roles of lncRNAs in trans-differentiation. Moreover, it is worth noting that the regulation of lncRNA showed obvious selection tendencies.

Among the upregulated genes that regulate lipogenesis, we noted the FABP family genes, which are key regulators for lipids synthesis and fat cell differentiation. The FABP family was first found in rat intestinal mucosa in 1972, including FABP3, FABP4, FABP5, and FABP7 [41]. Protein coding by FABP genes can bind to hydrophobic ligands of long-chain fatty acids, promote the transmembrane transport of plasma fatty acids to the synthesis sites of triglycerides or phospholipids in cells, and promote fat accumulation [42,43,44]. Exploring the changes in FABP family genes meant that we gained a deeper understanding of lipid synthesis and accumulation during the trans-differentiation of SMSCs. In this study, our RNA-seq and qPCR analyses showed that *FABP3*, *FABP4*, and *FABP5* were continuously expressed at high levels in the M and L stages of trans-differentiation. Additionally, the expression of FABP4 was higher than that of other FABP genes. This suggests that FABP genes, especially FABP4, can induce intracellular lipid droplet formation and accumulation at the late stages of SMSCs trans-differentiation. Meanwhile, we found that dozens of DE lncRNAs targeted three FABP genes through target gene prediction. These results provide useful information for the subsequent functional verification of candidate-regulated lncRNAs in muscle cells.

## 5. Conclusions

In conclusion, we gained insight into the time-series expression profiles of lncRNA and mRNA during the adipogenic transformation of porcine SMSCs through RNA sequencing. Thousands of lncRNAs and mRNAs showed significant changes on expressions during trans-differentiation. The key gene clusters that function for driving adipogenesis increased, and the genes in regulating cell cycle and DNA metabolism decreased, which was identified by using bioinformatics tools. In addition, we found that FABPs family genes, the key regulators of lipid synthesis and fat cell differentiation, have significant increases in expression levels during trans-differentiation, and these genes were controlled by dozens of lncRNAs. These findings provide a new set of potential drivers for muscle cell adipogenesis and also suggest that more attention should be directed to the evaluation of genetic programming and molecular interactions of trans-differentiation at different time stages.

## Data Availability

Raw sequencing data are available at the NCBI SRA (Sequence Read Archive) repository under accession number PRJNA820138.

## Supplementary Materials

The following are available online at https://www.mdpi.com/article/10.3390/cimb44050138/s1, **Figure S1**: Identification of porcine skeletal muscle satellite cells. Figure S2: The features of expression of long non-coding RNA (lncRNAs) and mRNAs. Table S1: Sequences of qPCR primer. Supplementary Table S2: Quality of samples sequencing data. Table S3: Top 20 highly expressed lncRNAs in the trans-differentiated cells. Table S4: Top 20 highly expressed mRNAs in the trans-differentiated cells. Table S5: The mRNAs with up-regulated expression in profiles 1, 2, and 11. **Table S6**: The mRNAs with down-regulated expression in profiles 4 and 7.
