# Supplementary material for: Time-Series Clustering of lncRNA-mRNA Expression during the Adipogenic Transdifferentiation of Porcine Skeletal Muscle Satellite Cells
Source: Curr Issues Mol Biol. 2022 May 6;44(5):2038–53. doi: 10.3390/cimb44050138 (PMC9164044; doi:10.3390/cimb44050138)
Supplement: Supplementary file 1 [file cimb-44-00138-s001.zip › Supplementary Table S1. Sequences of qPCR primer..pdf]

Supplementary Table S1. Sequences of qPCR primer.

Gene name	Forward	Reverse
GAPDH	CGACAGTCAGCCGCATCTT	CCAATACGACCAAATCCGTTG
FABP3	GCACCTTCAAGAGCACAGAGATCAG	TTGCCTCCATCCAGTGTCACAATG
FABP4	TGCAGAAGTGGGATGGAAAGACAAC	AATTCTGGTAGCCGTGACACCTTTC
FABP5	GGAGAGCACTTTGAAAACCACACAG	TCCCGTCCCATTCTTGATGTTGAAC
ENSSSCG00000044572	AGTCTAGGGCTGAGCAGGATGAAC	TGGGTCAGTTCCGAGCAGTAGC
ENSSSCG00000041875	TCTTAACGGTTTCACGCCCTCTTG	GGCACGAGACCGATAGTCAACAAG
ENSSSCG00000048856	GAGCGGCAGTTCGTGAAGATAGG	CATCGCACTGGCTTCCTCCTTC
ENSSSCG00000048556	TCCTTAGCTTGACGCCTTACCATTG	GACCTTGATGGACATGCACTCTCTC
ENSSSCG00000048719	TACTACATGCTTGAGGAGGGTGACG	GACTTGGCGGTGCTTCACATCC
ENSSSCG00000042841	ATCGGCTGGTAACGGCACATTG	CCACTTGGCTAGGAATGCTCAGTC
