# Supplementary material for: Time-Series Clustering of lncRNA-mRNA Expression during the Adipogenic Transdifferentiation of Porcine Skeletal Muscle Satellite Cells
Source: Curr Issues Mol Biol. 2022 May 6;44(5):2038–53. doi: 10.3390/cimb44050138 (PMC9164044; doi:10.3390/cimb44050138)
Supplement: Supplementary file 1 [file cimb-44-00138-s001.zip › Supplementary Table S2. Quality of samples sequencing data.pdf]

Supplementary Table S2. Quality of samples sequencing data.

Sample	Raw reads	Clean reads	Q20(%)	Q30(%)	Total mapped	Uniquely mapped
**P1**	73,952,334	72,892,958	98.55	95.41	70,312,240(96.46%)	65,770,497(90.23%)
**P2**	91,485,938	90,106,202	98.58	95.46	87,054,468(96.61%)	81,130,980(90.04%)
**P3**	73,933,702	72,802,744	98.49	95.22	70,285,299(96.54%)	65,000,525(89.28%)
**E1**	80,681,258	78,819,392	98.51	95.30	75,345,086(95.59%)	67,832,212(86.06%)
**E2**	90,524,772	89,119,066	98.56	95.41	85,277,131(95.69%)	77,022,248(86.43%)
**E3**	74,979,006	73,623,970	98.54	95.36	70,626,177(95.93%)	65,278,172(88.66%)
**M1**	79,624,626	78,338,782	98.58	95.47	75,499,073(96.38%)	69,527,291(88.75%)
**M2**	78,118,190	76,880,594	98.58	95.45	73,820,218(96.02%)	67,730,333(88.1%)
**M3**	74,280,636	73,073,728	98.61	95.54	70,022,671(95.82%)	64,260,470(87.94%)
**L1**	75,565,740	74,348,468	98.49	95.26	71,737,757(96.49%)	66,069,589(88.86%)
**L2**	84,452,358	83,110,374	98.48	95.18	80,369,523(96.7%)	72,907,409(87.72%)
**L3**	81,179,978	79,982,086	98.58	95.45	77,320,514(96.67%)	69,868,392(87.36%)
