# Supplementary material for: Time-Series Clustering of lncRNA-mRNA Expression during the Adipogenic Transdifferentiation of Porcine Skeletal Muscle Satellite Cells
Source: Curr Issues Mol Biol. 2022 May 6;44(5):2038–53. doi: 10.3390/cimb44050138 (PMC9164044; doi:10.3390/cimb44050138)
Supplement: Supplementary file 1 [file cimb-44-00138-s001.zip › Supplementary Table S3. Top 20 highly expressed lncRNAs in the trans-differentiated cells..pdf]

Supplementary Table S3. Top 20 highly expressed lncRNAs in the trans-differentiated cells.

Group P		Group E		Group M		Group L	
**Lnc RNA**	**TPM**	**Lnc RNA**	**TPM**	**Lnc RNA**	**TPM**	**Lnc RNA**	**TPM**
ENSSSCG00000051102	164,295	ENSSSCG00000051102	213,585	ENSSSCG00000051102	229,409	ENSSSCG00000051102	24,1178
ENSSSCG00000048719	5,591	ENSSSCG00000048719	12,222	ENSSSCG00000048719	13,414	ENSSSCG00000048719	24,125
ENSSSCG00000041875	2,745	ENSSSCG00000041875	10,748	ENSSSCG00000041875	5,874	MSTRG.23453	7,763
MSTRG.23453	1,600	MSTRG.23453	3,481	MSTRG.23453	4,361	ENSSSCG00000041875	6,445
ENSSSCG00000041401	1,050	ENSSSCG00000048856	1,590	ENSSSCG00000041401	1,793	ENSSSCG00000041401	1,643
ENSSSCG00000050447	613	ENSSSCG00000041401	1,513	ENSSSCG00000048856	794	ENSSSCG00000041596	721
ENSSSCG00000048856	452	ENSSSCG00000045913	903	ENSSSCG00000048556	571	ENSSSCG00000048556	645
MSTRG.8218	396	ENSSSCG00000041596	670	ENSSSCG00000045913	518	ENSSSCG00000045913	522
ENSSSCG00000042841	363	ENSSSCG00000048556	482	ENSSSCG00000050447	502	MSTRG.23454	448
MSTRG.23454	377	ENSSSCG00000050447	470	ENSSSCG00000041596	522	ENSSSCG00000048856	564
ENSSSCG00000041596	368	MSTRG.23454	289	MSTRG.23454	368	ENSSSCG00000050447	422
ENSSSCG00000048556	289	ENSSSCG00000044572	235	MSTRG.14725	236	ENSSSCG00000047453	249
MSTRG.14725	203	MSTRG.14725	244	ENSSSCG00000047453	213	MSTRG.14725	252
ENSSSCG00000047453	174	ENSSSCG00000047453	178	ENSSSCG00000042841	180	ENSSSCG00000042841	150
MSTRG.3929	177	ENSSSCG00000042841	201	ENSSSCG00000044572	154	ENSSSCG00000044572	140
ENSSSCG00000050649	115	ENSSSCG00000041461	114	MSTRG.8218	97	MSTRG.8218	121
ENSSSCG00000044572	107	MSTRG.8218	125	MSTRG.9920	68	ENSSSCG00000044397	65
ENSSSCG00000045913	134	ENSSSCG00000028322	50	ENSSSCG00000044841	68	ENSSSCG00000044841	59
ENSSSCG00000050010	119	MSTRG.3929	81	ENSSSCG00000044397	70	MSTRG.9920	51
MSTRG.20538	102	ENSSSCG00000044841	65	MSTRG.3929	57	ENSSSCG00000051070	42
