# Supplementary material for: Time-Series Clustering of lncRNA-mRNA Expression during the Adipogenic Transdifferentiation of Porcine Skeletal Muscle Satellite Cells
Source: Curr Issues Mol Biol. 2022 May 6;44(5):2038–53. doi: 10.3390/cimb44050138 (PMC9164044; doi:10.3390/cimb44050138)
Supplement: Supplementary file 1 [file cimb-44-00138-s001.zip › Supplementary Table S4. Top 20 mRNAs.pdf]

Supplementary Table S4. Top 20 highly expressed mRNAs in the trans-differentiated cells.

Group P		Group E		Group M		Group L	
**Gene ID**	**TPM**	**Gene ID**	**TPM**	**Gene ID**	**TPM**	**Gene ID**	**TPM**
ENSSSCG00000038507	457955.4	ENSSSCG00000038507	472273.4	ENSSSCG00000038507	477645.2	ENSSSCG00000038507	453478.6
ENSSSCG00000005009	80188.55	ENSSSCG00000005009	89902.57	ENSSSCG00000005009	90323.04	ENSSSCG00000005009	77273.13
ENSSSCG00000005314	53006.1	ENSSSCG00000005314	47790.76	ENSSSCG00000005314	56369.04	ENSSSCG00000005314	53962.13
ENSSSCG00000014725	14077.85	ENSSSCG00000014725	17823.94	ENSSSCG00000002144	18464.16	ENSSSCG00000002144	19911.62
ENSSSCG00000002144	13501.71	ENSSSCG00000002144	16979.15	ENSSSCG00000017645	17219.39	ENSSSCG00000014725	19369.38
ENSSSCG00000040470	11281.38	ENSSSCG00000040470	14770.68	ENSSSCG00000014725	16746.24	ENSSSCG00000017645	16471.2
ENSSSCG00000017645	10051.02	ENSSSCG00000017645	15193.53	ENSSSCG00000040470	11328.67	ENSSSCG00000040470	9310.41
ENSSSCG00000018082	6213.353	ENSSSCG00000004489	8894.39	ENSSSCG00000004489	6955.413	ENSSSCG00000018082	8075.21
ENSSSCG00000004489	6560.1	ENSSSCG00000018082	7027.52	ENSSSCG00000018082	6378.78	ENSSSCG00000018075	6472.417
ENSSSCG00000011033	6284.263	ENSSSCG00000035080	6538.77	ENSSSCG00000018075	5923.563	ENSSSCG00000040681	5631.86
ENSSSCG00000018078	5719.807	ENSSSCG00000004970	6660.04	ENSSSCG00000035080	5550.21	ENSSSCG00000004489	6004.333
ENSSSCG00000035080	5794.583	ENSSSCG00000018075	5612.77	ENSSSCG00000004970	5540.433	ENSSSCG00000035080	5277.197
ENSSSCG00000033854	5493.28	ENSSSCG00000028850	5153.783	ENSSSCG00000028850	4398.263	ENSSSCG00000036883	5237.673
ENSSSCG00000004970	5052.437	ENSSSCG00000032599	5214.243	ENSSSCG00000033310	4359.553	ENSSSCG00000028850	4501.457
ENSSSCG00000033310	4929.883	ENSSSCG00000033310	5327.717	ENSSSCG00000032599	4988.383	ENSSSCG00000018078	4397.773
ENSSSCG00000008245	4783.183	ENSSSCG00000039544	4229.073	ENSSSCG00000001502	3641.087	ENSSSCG00000004970	4850.763
ENSSSCG00000018075	4143.957	ENSSSCG00000001502	4551.607	ENSSSCG00000036883	3635.26	ENSSSCG00000032599	4930.747
ENSSSCG00000028850	4136.827	ENSSSCG00000020725	4241.977	ENSSSCG00000039544	3697.643	ENSSSCG00000039544	4099.197
ENSSSCG00000039544	4115.53	ENSSSCG00000018078	3621.16	ENSSSCG00000020725	3397.607	ENSSSCG00000033310	3757.107
ENSSSCG00000001502	3649.183	ENSSSCG00000035904	3884.213	ENSSSCG00000012842	3151.623	ENSSSCG00000001502	3036.48
