# Supplementary figures and images for: Time-Series Clustering of lncRNA-mRNA Expression during the Adipogenic Transdifferentiation of Porcine Skeletal Muscle Satellite Cells
Source: Curr Issues Mol Biol. 2022 May 6;44(5):2038–53. doi: 10.3390/cimb44050138 (PMC9164044; doi:10.3390/cimb44050138)

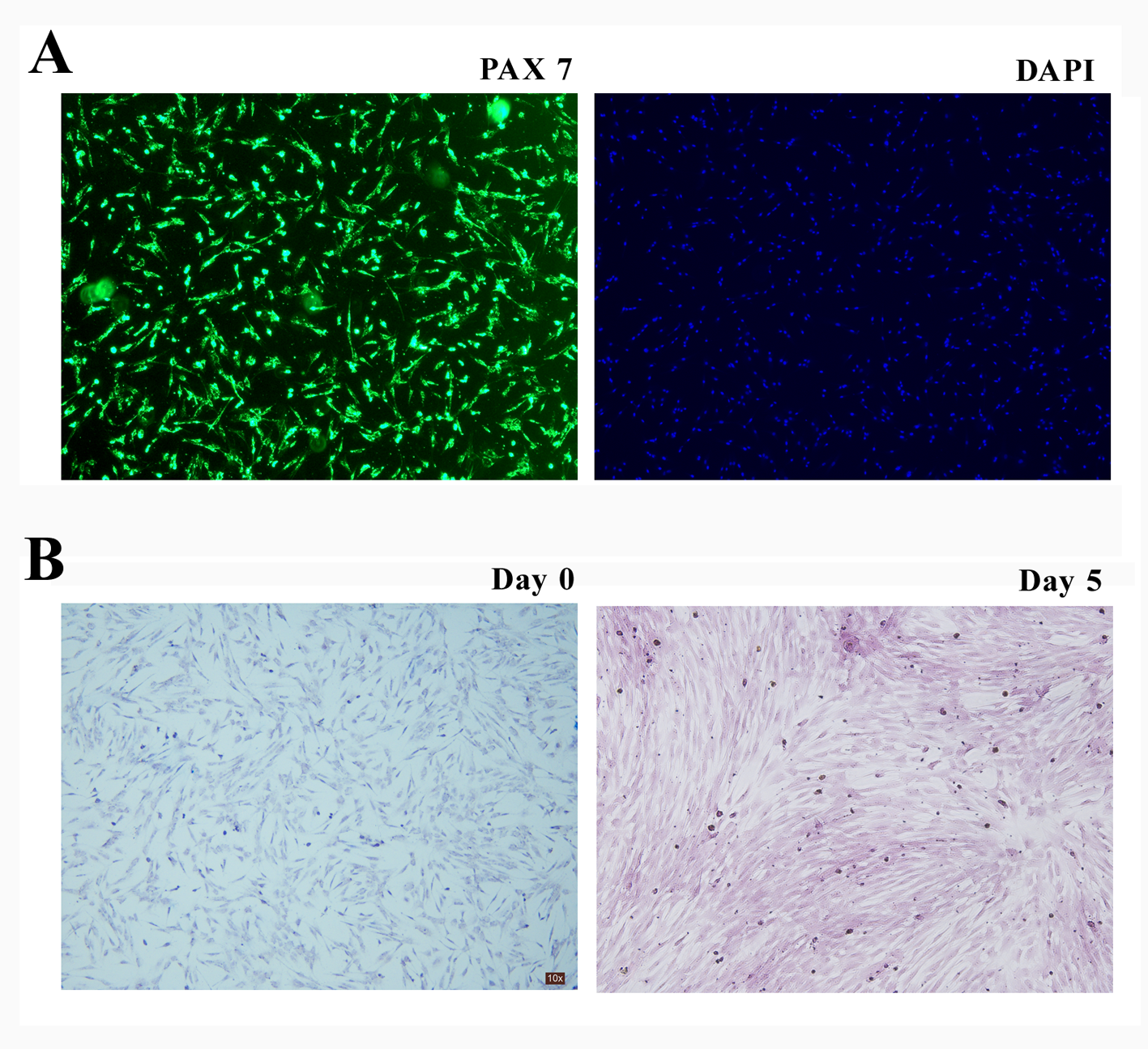

Supplement: Supplementary file 1 [file cimb-44-00138-s001.zip › Supplementary Figure S1.tif]

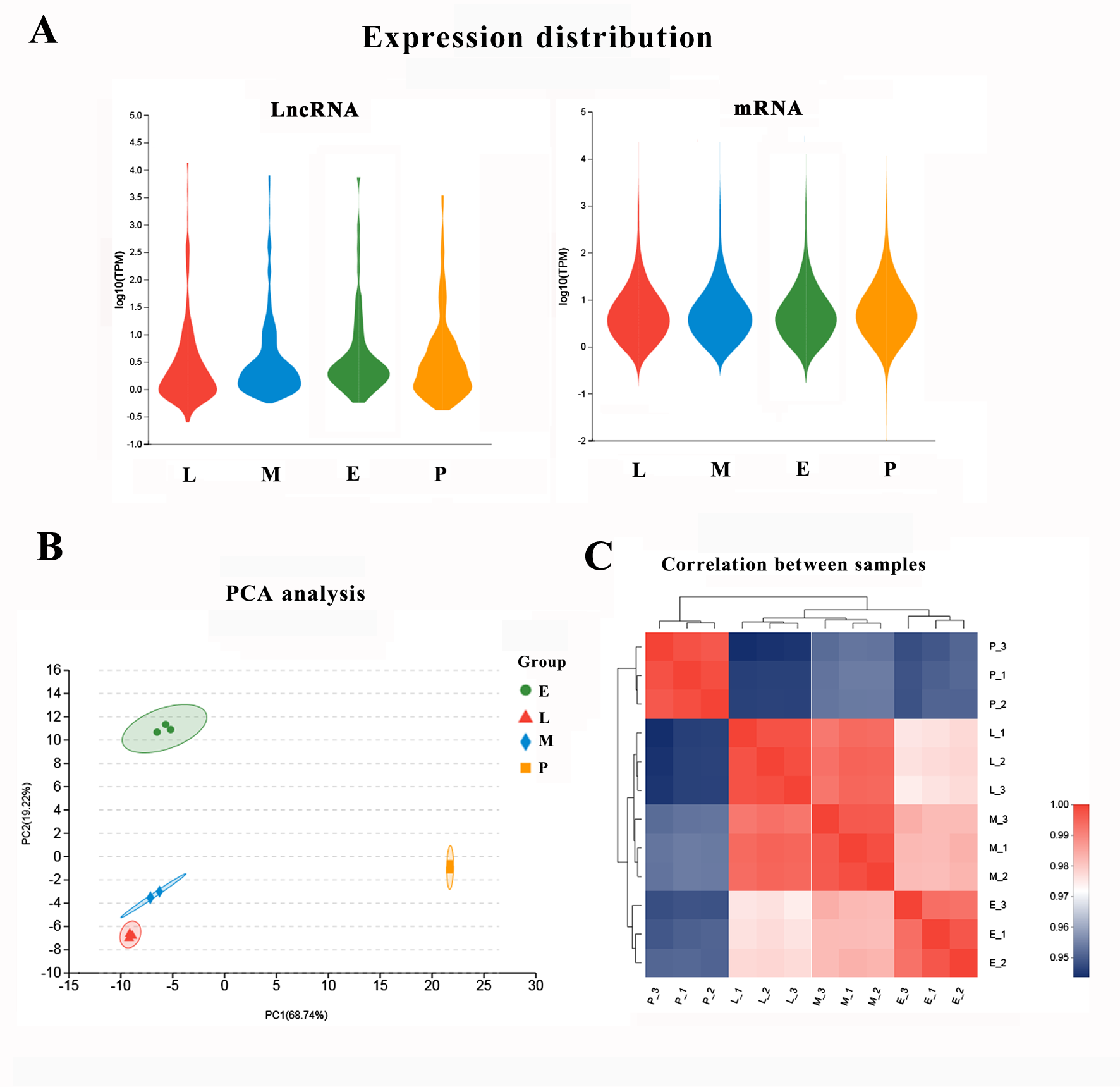

Supplement: Supplementary file 1 [file cimb-44-00138-s001.zip › Supplementary Figure S2.tif]
